# The association between serum soluble Klotho and chronic kidney disease among us adults ages 40 to 79 years: Cross-sectional study

**DOI:** 10.3389/fpubh.2022.995314

**Published:** 2022-10-06

**Authors:** Zilong Zhang, Xianghong Zhou, Linghui Deng, Kun Jin, Xingyu Xiong, Xingyang Su, Shi Qiu, Lu Yang

**Affiliations:** ^1^Department of Urology and Institute of Urology and National Clinical Research Center for Geriatrics, West China Hospital, Sichuan University, Chengdu, China; ^2^National Clinical Research Center of Geriatrics, The Center of Gerontology and Geriatrics, West China Hospital, Sichuan University, Chengdu, China; ^3^Department of Gerontology, West China Hospital of Sichuan University, Chengdu, China

**Keywords:** soluble α-Klotho, estimated glomerular filtration rate, chronic kidney disease, the national health and nutrition examination survey, cross-sectional study

## Abstract

**Background:**

Chronic kidney disease (CKD) is diagnosed in more than 26 million U.S. people, which increases the risk of many adverse events. α-Klotho was reported to have potential effects on kidney function. The purpose of this study was to investigated whether CKD prevalence is associated with α-Klotho levels in the U.S. people aged 40–79 years.

**Methods:**

Thirteen thousand five hundred eighty-nine participates in the National Health and Nutrition Examination Survey 2007–2016 aged 40–79 with information of Klotho and kidney function were included. The association between CKD and Klotho was calculated using multivariate linear or logistic regression models with adjustment of several possibly confounding variables. Subgroup analyses stratified by age, BMI, and diabetes mellitus were conducted. The non-linear relationship between Klotho and dependent variables with a non-normality of residues was assessed using smooth curve fitting and the segmented regression (also known as piece-wise regression) models.

**Results:**

Among 13,589 participants, the median of Klotho levels was 803.10 pg/mL, mean eGFR of all participants was 86.96 (SD = 19.88) mL/min/1.73 m^2^, and CKD was diagnosed in 20.11% of them (*N* = 2733). In the fully adjusted model, eGFR was positively associated with Klotho (*β* = 5.14, 95%CI 4.13-6.15, *p* < 0.001), while CKD was negatively associated with Klotho (stage ≧ 1, OR = 0.62, 95% CI 0.50–0.76, *p* < 0.001; stage ≧ 3, OR = 0.31, 95% CI 0.24–0.41, *p* < 0.001). The non-linear relationship showed that occurrence of CKD stage> 1 and albuminuria were negatively associated with Klotho when Klotho smaller than turning point (for whether CKD stage> 1, turning point *K* = 6.85, Klotho < *K*, OR = 0.44, *p* < 0.001; for albuminuria, turning point *K* = 6.84, Klotho < *K*, OR = 0.59, *p* < 0.001).

**Conclusion:**

Serum soluble Klotho levels were positively associated with eGFR and negatively associated with the prevalence of CKD, especially in elderly, obese, and diabetic patients.

## Introduction

Chronic kidney disease (CKD) is defined as abnormal kidney structures and function that affects health and lasts for at least 3 months (1). According toprevious literature, CKD increases the risk of cardiovascular disease and overall mortality, bringing about greater social and economic burdens (2, 3). In the United States, CKD was diagnosed among more than 26 million adults, characterized by the decrease in glomerular filtration rate (GFR) and the increase in urinary albumin (4).

*Klotho* is an aging suppressor gene firstly reported by Kuro-o et al. in 1997, which was named after one of the Fates, the Greek goddess of the thread of life (5). α-Klotho (referred to as Klotho hereafter), one of the *Klotho* gene products, is a single-pass transmembrane protein consisting of 1012 amino acid, containing two long extracellular domains and one short intracellular domain. The extracellular part of α-Klotho can be cut off by membrane proteases, and a soluble form is than generated (6). Klotho is mostly expressed in the distal convoluted tubules, as well as the choroid plexus and the parathyroid glands, functioning as a co-receptor of FGF-23 (7, 8). Previous researches have demonstrated that Klotho has pathophysiological association with various aging-related disorders, including atrophy of multiple organs, premature atherosclerosis, vascular and soft tissue calcification and cancer (6, 9).

The relationship between Klotho and CKD was well reported by genetic studies, which was explained by renal fibrosis (10), inflammation (11), and the vascular calcification (12). However, different results were shown in several cross-sectional and prospective cohort studies. One previous cohort study with 2,496 participants aged 70–79 analyzed the relationship between Klotho and incident CKD, as well as the interaction between Klotho and FGF-23 or phosphorus, demonstrating the association between low serum Klotho levels and the decline in kidney function, while another study demonstrated a different result that Klotho levels increased in patients with worse kidney function (13, 14).

The purpose of the present study is to validate the relationship between Klotho and CKD using a relatively large and national representative population aged 40–79 from the United States. We hypothesized that the kidney function declined as the serum Klotho level decreases.

## Materials and methods

### Population and study design

The National Health and Nutrition Examination Survey (NHANES) is a cross-sectional study conducted by the National Center for Health Statistics (NCHS), which is designed to examine the overall health and nutrition status of a nationally representative, non-institutional population in the United States. The survey was conducted every 2 years, with demographic, physical, laboratory, questionnaire, and dietary information collected in each cycle.

For the present study, all participants from five NHANES cycles from 2007 to 2016 were included. To investigate the association between Klotho and kidney function, the relative information was indispensable. Thus, participants as follows were excluded: (a) participants with missing data of serum soluble Klotho; (b) participants without data to estimate GFR, including serum creatinine, age, gender, and race; (c) participants without data of urine creatinine or urine albumin.

**Figure 1:**
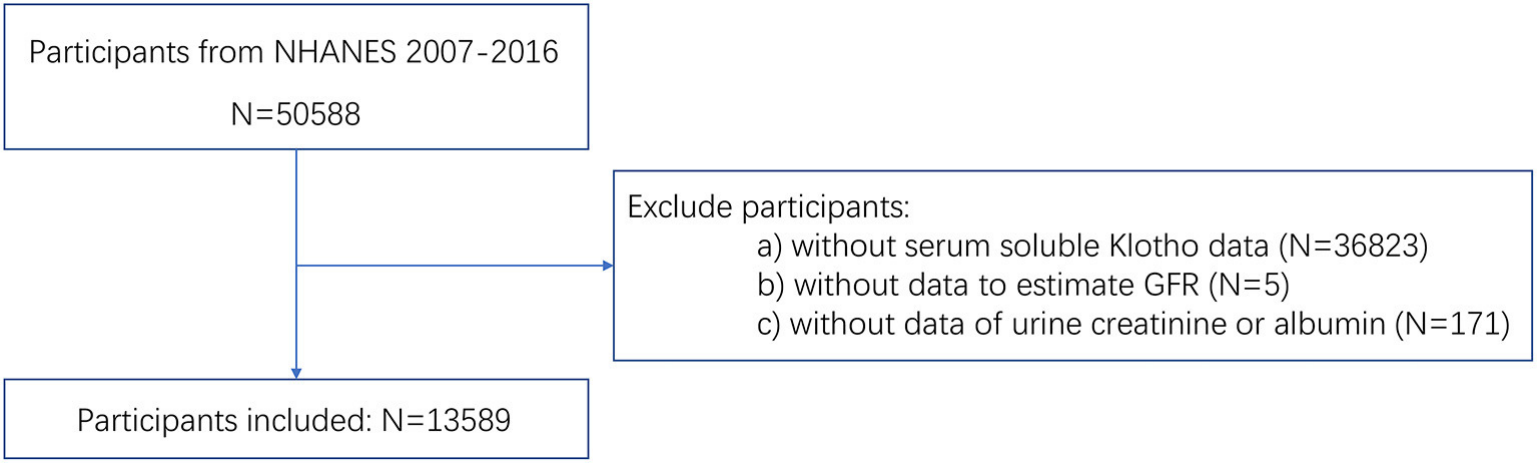
Flowchart of participants selection.

A total of 50,588 people participated in NHANES from 2007 to 2016. Among them, 36,999 people were excluded according to the exclusion criteria. Finally, a total of 13,589 participants were enrolled in our study. The participants selection flowchart was shown in Figure 1.

The study was approved by the NCHS Ethics Review Board, and written informed consents were signed by all participants. All detail information is publicly available at https://www.cdc.gov/nchs/nhanes/.

### Measurement of serum soluble Klotho

The serum samples were collected in the mobile examination center and stored in the −80°C until they were analyzed. The Klotho measurement was performed in the Northwest Lipid Metabolism and Diabetes Research Laboratories, Division of Metabolism, Endocrinology, and Nutrition, University of Washington, using a commercial ELISA kit produced by IBL International, Japan. Each sample was analyzed in duplication and the average of 2 concentrations was used as the final value of the sample. If the values of duplication exceeded 10%, the sample would be measured repeatedly. Two samples with low and high Klotho concentration were analyzed in each ELISA plate for quality control, and if the results were not within 2 standard deviations of assigned value, the entire analysis was rejected and to be repeated. The IBL ELISA method for measurement of Klotho concentration in human samples was further discussed by the NHANES (shown in Supplementary File). The details about laboratory methodology as well as the quality assurance and monitoring are available at https://wwwn.cdc.gov/Nchs/Nhanes/2007-2008/SSKL_E.htm.

### Assessment of chronic kidney disease

The estimated GFR (eGFR) was calculated using the Chronic Kidney Disease Epidemiology Collaboration (CKD-EPI) equation:


$$\begin{array}{l} eGFR\;=\;141\;\times \;\min{\;(\frac{Scr}{\kappa },\;1)}^{\alpha }\;\times \;\max{\;(\frac{Scr}{\kappa },\;1)}^{-1.209}\\ \;\times \;0.993^{Age}\;\times \;1.018\;[if\;female]\;\times \;1.159\;[if\;black], \end{array}$$

in which Scr is standardized serum creatinine in mg/dL, *κ* is 0.7 for females and 0.9 for males, α is −0.329 for females and −0.411 for males, min means the minimum of Scr/*κ* or 1, and max means maximum of Scr/*κ* or 1. The eGFR units are mL/min/1.73 m^2^ (15). Urinary albumin-creatinine ratio (ACR) was calculated using equation: ACR (mg/g) = urinary albumin (mg/dL)/urinary creatinine (g/dL). The kidney damage was indicated by the presence of albuminuria, which was defined as ACR ≧ 30 mg/g. The CKD stages were defined with eGFR and/or evidence of kidney damage according to the KDIGO guidelines: stage 1, eGFR ≧ 90 mL/min/1.73 m^2^ with ACR ≧ 30 mg/g; stage 2, eGFR 60–89 mL/min/1.73 m^2^ with ACR ≧ 30 mg/g; stage 3, eGFR 30–59 mL/min/1.73 m^2^; stage 4, eGFR 15–29 mL/min/1.73 m^2^; stage 5, eGFR < 15 mL/min/1.73 m^2^ (16).

### Covariates

The demographics variables included age, race (categorical, Mexican American, Other Hispanic, Non-Hispanic White, Non-Hispanic Black, and Others), education level (categorical, less than high school, high school or general educational development, above high school), marital status (categorical, married or living with partner, living alone), and the ratio of income-poverty (categorical, ≦ 1.3 as low, 1.3–3.5 as middle, > 3.5 as high). The ratio of income-poverty was calculated by dividing family income by the poverty guidelines, specific to family size, as well as the appropriate year and state. The examination variables was body mass index (BMI) (categorical, ≦ 25 as normal weight 25–30 as over-weight, > 30 as obese), which was calculated by dividing weight by height squared. The laboratory data included serum calcium and serum phosphorus. The questionnaire variables contained smoking status (categorical, less than 100 in entire life as never, 100 or more but no smoking now as former, 100 or more with smoking now as current), alcohol consumption (categorical, 0 g/day as none, 0.1–27.9 g/day for men and 0.1–13.9 g/day for women as moderate, 28.0 g or more/day for man and 14.0 g or more/day for women as heavy), physical activity (categorical, less than moderate, moderate, and vigorous), and CAD score (categorical, 0 as none, 1 as slight, 2–5 as heavy). The score was generated by accumulated points for following rules. Participants got 1 point for each of hypertension, history of transient ischemic attack or stroke, and coronary artery disease. One point was added if they had impaired glucose tolerance, and 2 points for the diagnosis of diabetes. Diabetes was defined as those who were told by doctor that they had “diabetes or sugar diabetes” when they were not pregnant, were currently using insulin or oral hypoglycemic drugs, or had a glycohaemoglobin> 6.5%.

### Statistics

Missing values of the covariates were replaced by the median of the continuous variables and the mode of the categorical variables. In the present study, normal continuous variables were presented with mean (SD), while non-normal continuous variables were presented with median (lower quartile, upper quartile). Categorical variables were described using numbers (percentage). The differences between characteristics of participants in four groups divided by Klotho quartiles, age quartiles, gender, and race were calculated with Kruskal-Wallis H test for continuous variables and chi-square test for categorical variables. If the categorical variables had a theoretical number less than 10%, Fisher's precision probability test were applied.

To validate the association between Klotho and CKD, we selected eGFR, CKD (defined as CKD stage ≧ 1 or CKD stage ≧ 3), and albuminuria (defined as ACR ≧ 30mg/g) as dependent variables. Serum Klotho concentrations were logarithmic transformed due to its skew distribution. Linear regression and logistic regression were performed. Multivariable linear regression was used when evaluating the associations between Klotho and eGFR, and multivariable logistic regression was applied when analyzing the association between Klotho and CKD or albuminuria. In each regression, 3 models were established: Model I, a non-adjusted crude model; Model II, adjusted with age, gender, race, ration of income-poverty, education level, and marital status; Model III, Model II plus BMI, CAD score, smoke status, alcohol intake, physical activity, serum calcium, and serum phosphorus. The QQ plots of residues were presented to assess the normality of residues in all regression. To simply describe the non-linear relationship between Klotho and dependent variables with a non-normality of residues, we applied smooth curve fitting and the segmented regression (also known as piece-wise regression) models. Log-likelihood ratio test comparing one-line (non-segmented) model with segmented regression model was also conducted.

Stratified analyses were performed according to age, BMI, and diabetes, while interaction analyses on these factors were also performed with the log likelihood ratio test. The subgroup analysis was performed in Model III with all covariates adjusted except the stratification factor itself. The associations between Klotho and eGFR among people in different CKD stages (including no CKD population) were assessed using multivariable linear regression with all covariates adjusted.

Sensitive analyses were conducted by calculating the eGFR using the Modification of Diet in Renal Disease (MDRD) equation:


$$\begin{array}{l} eGFR\;=\;175\;\times \;Scr^{-1.154}\;\times age^{-0.203}\;\times \;0.742[if\;female]\\ \;\times \;1.212\;[if\;black]. \end{array}$$

According to the NHANES Survey Methods and Analytic Guidelines, recommended weighting methodology was applied during the entire process. All analyses were conducted by the software package R (www.R-project.org, The R Foundation) and EmpowerStats (www.empowerstats.com, X&Y Solution, Inc). The *p*-values of multiple testing were all adjusted and two-tailed *p* < 0.05 was considered as statistically significant.

## Results

### Baseline characteristic of participants

Table 1 showed the baseline characteristics of the participants. Among all subjects, the mean age was 57.64 (SD = 10.84), with 6,578 (48.41%) males. The median of Klotho levels was 803.10 (Q1 = 655.60 and Q3 = 994.40) pg/mL. The mean eGFR of all participants was 86.96 (SD = 19.88) mL/min/1.73m^2^, and CKD was diagnosed in 20.11% of them (*N* = 2733, 740 in stage 1, 715 in stage 2, 1,146 in stage 3, 93 in stage 4, and 39 in stage 5). Differences between age, gender, race, education level, BMI, CAD score, diabetes, smoking status, alcohol consumption, serum calcium, eGFR, albuminuria and CKD prevalence were observed with statistical significance. People with lower Klotho levels were more likely to be older, male, over-weight or obese, with diabetic, and with higher CAD score or lower serum calcium concentration. More detailed information about the baseline characteristics were presented in Supplementary Tables 1–4.

**Table 1:** Baseline characteristics of participants^a^.

**Number of subjects**	**13,589**
Age	57.64 (10.84)
**Gender**	
Male	6,578 (48.41%)
Female	7,011 (51.59%)
**Race**	
Mexican American	2,164 (15.92%)
Other Hispanic	1,563 (11.50%)
Non-Hispanic White	5,842 (42.99%)
Non-Hispanic Black	2,679 (19.71%)
Other Race	1,341 (9.87%)
**BMI**	
Normal weight	3,215 (23.89%)
Over-weight	4,711 (35.01%)
Obese	5,532 (41.11%)
**CAD score**	
None	5,838 (44.40%)
Slight	3,939 (29.95%)
Heavy	3,373 (25.65%)
**Diabetes**	
No	10,647(78.35%)
Yes	2,942 (21.65%)

A normal continuous variables were presented with mean (SD), while non-normal continuous variables were presented with median (lower quartile, upper quartile). Categorical variables were described using numbers (percentage).

*BMI*, body mass index; *CAD score*, the cardiovascular disease score; *eGFR*, estimated glomerular filtration rate; *ACR*, albumin-creatinine ratio; *CKD*, chronic kidney disease.

### Association between Klotho and CKD

**Figure 2:**
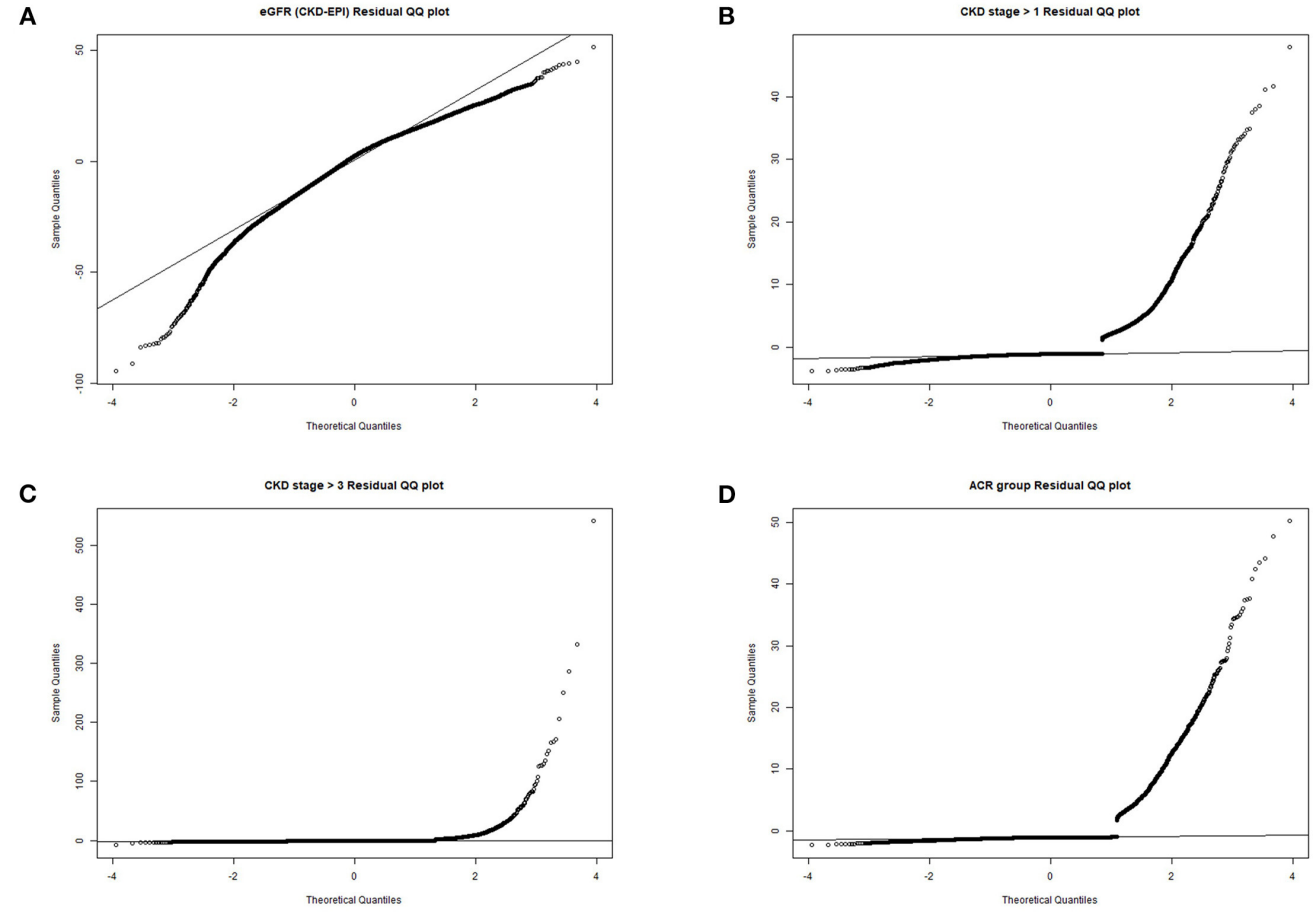
QQ plots of residues. **(A)** eGFR residual QQ plot; **(B)** CKD stage> 1 residual QQ plot; **(C)** CKD stage> 3 residual QQ plot; **(D)** albuminuria residual QQ plot.

Table 2 presented the results on the association between Klotho and CKD. In non-adjusted crude model, when log-transformed Klotho concentration increased 1 unit, eGFR was 7.77 mL/min/1.73m^2^ higher (*p* < 0.001), and the risk of CKD was lower (stage ≧ 1 decreased 47%, and stage ≧ 3 decreased 72%, both of *p-*values < 0.001). Albuminuria risk was also lower with increasing Klotho levels (OR = 0.77, 95%CI 0.61–0.97, *p* = 0.027). Moreover, with the adjustment of all covariates in Model II, similar results were also observed. eGFR was positively associated with Klotho levels (*β* = 5.14, 95%CI 4.13–6.15, *p* < 0.001), while CKD risk was negatively correlated (stage ≧ 1, OR = 0.62, 95% CI 0.50–0.76, *p* < 0.001; stage ≧ 3, OR = 0.31, 95% CI 0.24–0.41, *p* < 0.001). However, the association between Klotho and albuminuria risk was not significant anymore (OR = 0.90, 95% CI 0.71–1.15, *p* = 0.411). The residues QQ plots of all regressions were presented in Figure 2. For associations between Klotho and all three categorical dependent variables (CKD > stage 1, CKD > stage 3, and albuminuria), the QQ plot showed a non-normality of residues.

**Table 2:** Association between serum soluble Klotho and CKD.

	**Number of subjects**	**Crude model**		**Model I^a^**		**Model II^b^**	
		**β/OR, 95%CI**	***p*** **-values**	**β/OR, 95%CI**	***p*** **-values**	**β/OR, 95%CI**	***p*-values**
eGFR, mL/min/1.73m^2^	13589	7.77 (6.58,8.95)	<0.001	4.85 (3.88,5.81)	<0.001	5.14 (4.13,6.15)	<0.001
CKD stage ≧ 1	2733/13589	0.53 (0.44,0.65)	<0.001	0.61 (0.50,0.75)	<0.001	0.62 (0.50,0.76)	<0.001
CKD stage ≧ 3	1278/13589	0.28 (0.22,0.36)	<0.001	0.33 (0.25,0.43)	<0.001	0.31 (0.24,0.41)	<0.001
ACR ≧ 30 mg/g	1901/13589	0.77 (0.61,0.97)	0.027	0.85 (0.67,1.07)	0.17	0.90 (0.71,1.15)	0.411

a Model I: adjusted for age, gender, race, ratio of income-poverty, educational levels, and marital status.

b Model II: adjusted for age, gender, race, ratio of income-poverty, educational levels, marital status, BMI, CAD score, smoking, alcohol, physical activity, serum calcium, and serum phosphorus.

*OR*, odds ration; 95% CI, 95% confidential interval; *eGFR*, estimated glomerular filtration rate; *CKD*, chronic kidney disease; *ACR*, urinary albumin-creatinine ratio.

**Figure 3:**
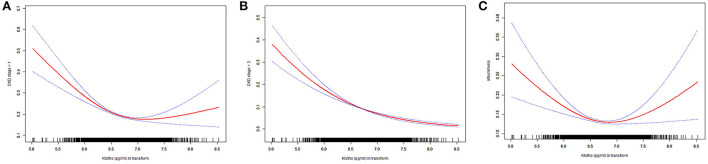
Smooth curve fitting. **(A)** association between Klotho ln transform and whether CKD stage> 1; **(B)** association between Klotho ln transform and whether CKD stage > 3; **(C)** association between Klotho ln transform and albuminuria.

The smooth curves of association between Klotho and above three dependent variables were presented in Figure 3. From the curves, obvious turning points were observed in associations between Klotho and dependent variables whether CKD stage > 1 and albuminuria. The segment regression models were presented in Table 3. The results showed that both the occurrence of CKD stage > 1 and albuminuria were negatively associated with Klotho when Klotho smaller than turning point (for whether CKD stage > 1, turning point *K* = 6.85, Klotho < *K*, OR = 0.44, *p* < 0.001; for albuminuria, turning point *K* = 6.84, Klotho < *K*, OR = 0.59, *p* < 0.001).

**Table 3:** Threshold effect analysis for the association between serum Klotho concentrations and two categorical dependent variables: Whether CKD stage> 1 and albuminuria^a^.

**Models**	**Whether CKD stage**> **1**		**Albuminuria**	
	**OR (95% CI)**	***p-*** **values**	**OR (95% CI)**	***p-*** **values**
**Model I**				
One line slop	0.62 (0.50, 0.72)	<0.001	0.90 (0.71, 1.15)	0.411
**Model II**				
Turning point (*K*)^a^	6.85	–	6.84	–
< *K* slope 1	0.44 (0.36, 0.54)	<0.001	0.59 (0.47, 0.74)	<0.001
> *K* slope 2	1.41 (1.00, 1.99)	0.051	1.77 (1.23, 2.53)	0.002
Slope 2–slope 1	3.21 (2.01, 5.12)	<0.001	3.00 (1.82, 4.95)	<0.001
Predicted at *K*	−1.67 (−1.75, −1.60)	–	−2.07 (−2.15, −1.98)	–
Log likelihood ratio test	–	<0.001	–	<0.001

Model I, linear model.

Model II, non-linear model.

Log likelihood ratio test: *p*-value < 0.05 means Model II is significantly different from Model I, which indicates a non-linear relationship.

a Adjusted for age, gender, race, ratio of income-poverty, educational levels, marital status, BMI, CAD score, smoking, alcohol, physical activity, serum calcium, and serum phosphorus.

The associations between Klotho and eGFR among people in different CKD stages were analyzed. We found that the significant associations can be observed in group without CKD (OR = 2.02, 95% CI 1.26–2.78, *p* < 0.001), with CKD stage 2 (OR = 2.87, 95% CI 0.94–4.79, *p* = 0.004), and with CKD stage 3 (OR = 2.03, 95% CI 0.62–3.44, *p* = 0.005). In other groups, the associations were not significant (shown in Supplementary Table 5).

In order to further investigate the relationship between Klotho levels and kidney function, we performed sensitivity analysis through recalculating eGFR using MDRD equation. In fully adjusted model, the association was positive between Klotho and eGFR (*β* = 5.91, 95% CI 4.63–7.18, *p* < 0.001), and negative between Klotho and both of two stage of CKD (stage ≧ 1, OR = 0.59, 95% CI 0.48–0.72, *p* < 0.001; stage ≧ 3, OR = 0.34, 95% CI 0.26–0.44, *p* < 0.001). The sensitive analysis results supported the main results described above (shown in Supplementary Table 6).

### Subgroup analysis

**Figure 4:**
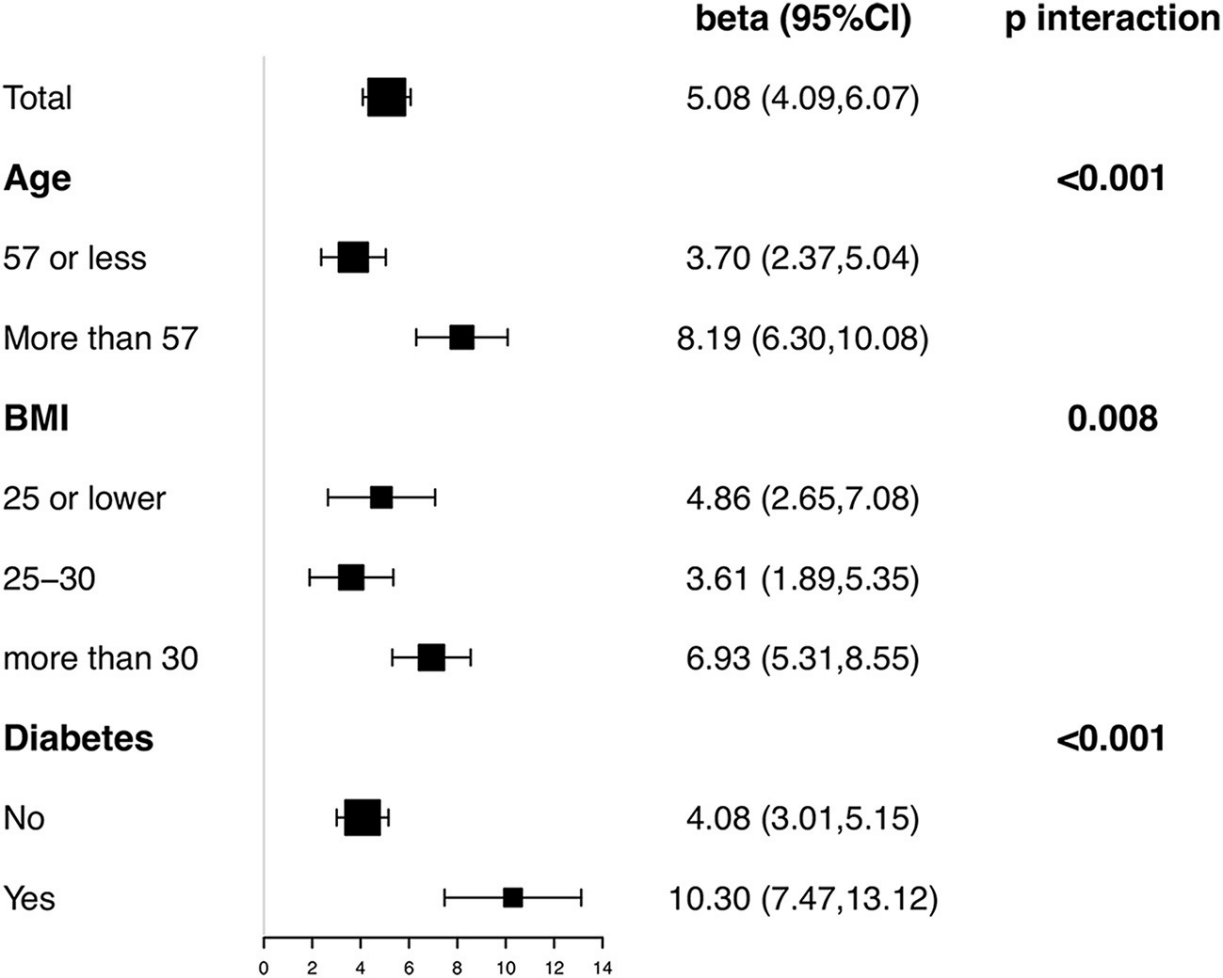
subgroup analysis on association between serum soluble Klotho and CKD.

Stratified and interaction analyses were conducted according to age, BMI, and diabetes. Figure 4 showed the results on association between Klotho and eGFR in each subgroup with all covariates in Model III adjusted except the stratification factor itself. Significantly positive association was observed in all subgroups. Besides, interaction test was significant for all three factors (*p* < 0.001 for age, *p* = 0.008 for BMI, and *p* < 0.001 for diabetes). In participants> 57 years old, obese, or with diabetes, the association between eGFR and Klotho was detected with a higher effect size.

## Discussion

The present study examined the relationship between serum soluble Klotho and CKD among 4–9 years old people in the United States. As a cross-sectional study, this study enrolled totally 13,589 participants from NHANES 2007–2016. The results reported that an elevated serum soluble Klotho level was associated with the increase of eGFR and the decrease of the occurrence of CKD stage> 3. The same result existed after adjusting covariates or recalculating eGFR with MDRD equation. The occurrence of CKD stage> 1 and albuminuria were negatively associated with Klotho when Klotho smaller than turning point.

According to the results of this study, reduced Klotho levels were associated with an increased risk of CKD. Previous studies have reported similar results. Drew et al. conducted a cohort study with participants aged 70–79, in which CKD was only defined with eGFR less than 60 mL/min/1.73 m^2^. The results presented an inverse relationship between Klotho and CKD prevalence, although not statistically significantly (13). In the present study, we used eGFR and albuminuria as the evidence of kidney damage together to define CKD. The reason is that in some patients with albuminuria, EGFR has not decreased, even though their kidneys have been damaged (patients with stage 1 or 2 CKD) (17).

Another research conducted by Alexander Scholze et al. was a randomized controlled trial with a follow-up of 8 weeks. This study demonstrated that among CKD patients with low FGF-23 levels, a decrease in Klotho leads to a decrease in eGFR (18). The current study aimed to explore the association of Klotho with eGFR in the entire population with and without CKD, and whether Klotho is associated with the prevalence of CKD, thus not excluding the normal population without CKD.

However, not all previous evidence is inconsistent with this study. Sarah Seiler et al. reported a cross-sectional study with 321 participants enrolled in, which presented that Klotho was not associated with kidney function (19). But they only recruited patients with CKD stage 2 to stage 4, which resulted in a difference in participants' soluble Klotho levels between Sarah Seiler et al. 's study and the present study (538 pg/mL vs. 803.10 pg/mL). It is reasonable to assume that the different baseline characteristics of the study populations may account for the observed differences in results. Another cross-sectional study conducted by Sridevi Devaraj et al. demonstrated that patients with CKD had higher Klotho levels, in which CKD was defined as creatinine greater than 2mg/dL over 12 months. While in our present study, CKD-EPI formula was used to calculate eGFR according to KDIGO guidelines, including age, sex, race, creatinine and other information, and evidence of kidney damage was also used to define CKD.

The mechanism of Klotho's renoprotective effect on CKD has been reported in many previous studies. Animal experiments showed that a Klotho genetic manipulation could ameliorate the renal functions of mice with progressive renal injury (20). Doi et al. observed that renal fibrosis was related to Klotho deficiency in mice, and demonstrated that the fibrosis-associated renal change could be prevented by Klotho treatment (10). Also, Zhao et al. reported that Klotho depletion led to increased inflammation of kidney *via* RelA (Serine)^536^ phosphorylation (11). Besides, another study presented that Klotho could ameliorate vascular calcification (12).

As a cross-sectional study, the present study can be explained differently. According to previous studies, Klotho is mainly expressed in the distal convoluted tubules, and kidney acts as a principal organ in mediating the expression of Klotho (21, 22). Thus, the pathological status of CKD affects the physiological function of kidney, which down-regulated the expression and production of Klotho (23).

It is worth noting that in different CKD stages, the relationship between Klotho and eGFR is different. In general, we think the associations were more significant in population without CKD and with lower CKD stages than higher CKD stages. However, due to the large variation in sample size and baseline characteristics among populations with different CKD stages, the results still need to be validated by further studies, including large cohorts and basic experiments.

There are some strengths of the present study. First, this cross-sectional study had 13,589 participants from NHANES enrolled in. By applying recommended weighting methodology according to the NHANES Survey Methods and Analytic Guidelines, the population has national representation. Second, the present study used multiple outcomes to represent kidney function of CKD. Instead of using eGFR < 60 mL/min/1.73 m^2^ to define CKD, this study used a combination of eGFR and albuminuria to define CKD, considering the patients with albuminuria but without decreased eGFR. Finally, subgroup analysis stratified by diabetes mellitus were performed and interaction terms on diabetes and Klotho on CKD were investigated, because Previous studies have suggested that Klotho deficiency is associated with the rapid progression of renal function decline in diabetic patients (24).

However, several limitations in the present study are discussed. As mentioned above, this study could not establish a causal relationship between Klotho and CKD because it was a cross-sectional study. In addition, there may still be confounding factors that we have not adjusted for. Furthermore, the study did not follow up the dynamic changes of renal function in patients with CKD. It is hoped that future studies of higher quality will focus on Klotho levels and CKD and renal function, so as to explore its potential clinical value.

## Conclusion

Serum soluble Klotho levels were positively associated with eGFR and inversely associated with the prevalence of CKD stage 3 or more. Dose-response relationships were observed between Klotho and the occurrence of CKD stage> 1 and albuminuria.

## Data availability statement

The datasets presented in this study can be found in online repositories. The names of the repository/repositories and accession number(s) can be found below: https://www.cdc.gov/nchs/nhanes.

## Ethics statement

The studies involving human participants were reviewed and approved by the National Center for Health Statistics Ethics Review Board. The patients/participants provided their written informed consent to participate in this study.

## Author contributions

ZZ: data collection, methodology, and writing. XZ: data collection and table and figure preparation. LD: methodology, formal analysis, and writing. KJ: methodology. XX and XS: data collection. SQ and LY: conceptualization, methodology, and reviewing. All authors contributed to the article and approved the submitted version.

## Funding

The work was supported by the National key research and development program of China (Grant No. 2017YFC0908003), National Natural Science Foundation of China (Grant Nos. 81902578 and 81974098), China Post-docotral Science Foundation (2017M612971), Post-doctoral Science Research Foundation of Sichuan University (2020SCU12041), Post-Doctor Research Project, West China Hospital, Sichuan University (2018HYBH085), National Clinical Research Center for Geriatrics, West China Hospital, Sichuan University (Z2018C01).

## Conflict of interest

The authors declare that the research was conducted in the absence of any commercial or financial relationships that could be construed as a potential conflict of interest.

## Publisher's note

All claims expressed in this article are solely those of the authors and do not necessarily represent those of their affiliated organizations, or those of the publisher, the editors and the reviewers. Any product that may be evaluated in this article, or claim that may be made by its manufacturer, is not guaranteed or endorsed by the publisher.
