# Supplementary material for: The association between serum soluble Klotho and chronic kidney disease among us adults ages 40 to 79 years: Cross-sectional study
Source: Front Public Health. 2022 Oct 6;10:995314. doi: 10.3389/fpubh.2022.995314 (PMC9582855; doi:10.3389/fpubh.2022.995314)
Supplement: Supplementary file 1 [file Table_1.docx]

Supplementary Material

Description of Laboratory Methodology in Measurement of Klotho Concentration in Human Samples with the IBL ELISA Kit

The Northwest Lipid Metabolism and Diabetes Research Laboratories, Division of Metabolism, Endocrinology, and Nutrition, University of Washington, performed analyses on all but four fresh-frozen (pristine) samples received from the Centers for Disease Control and Prevention. Prior to the start of the study, an extensive validation of the IBL ELISA method for measurement of Klotho concentration in human samples was performed and the results of the Validation provided to the study investigators.

In summary, 1) the assay standard curves and the relative signals of the calibrator concentrations were consistently within the criteria specified by the manufacturer, 2) the obtained assay sensitivity was calculated to be 4.33 pg/mL while the manufacturer claimed sensitivity was 6.15 pg/mL, 3) two samples with very high and high Klotho concentrations were used at different dilution to evaluate the assay linearity. Plots of the expected vs. the obtained values, demonstrated an excellent linearity in the assay measurement range (R2 = 0.998 and 0.997, respectively), 4) the intra-assay precision obtained on two recombinant Klotho samples and two human samples exhibited a coefficient of variation of 3.2% and 3.9% for the recombinant and 2.3% and 3.3% for the human samples, 5) The same samples analyzed in duplicate over 4 different days, showed an inter-assay CV of 2.8% and 3.5% for the recombinant samples and 3.8% and 3.4% for the human samples, 5) the reference ranges were evaluated in 114 samples from apparently healthy donors and the alpha-Klotho levels ranged from 285.8 to 1638.6 pg/mL with a mean of 698.0 pg/mL.

All sample analyses were performed in duplicate according to the manufacturer’s protocol and all the results were checked to meet the laboratory’s standardized criteria for acceptability prior to being released for reporting.

Supplementary Table 1, Baseline Characteristics of Participants^a^

|  | Total | Klotho groups | | | | p value^b^ |
| --- | --- | --- | --- | --- | --- | --- |
|  |  | Q1 | Q2 | Q3 | Q4 |  |
| Number of subjects | 13589 | 3397 | 3395 | 3399 | 3398 |  |
| Age | 57.64 (10.84) | 59.02 (11.09) | 57.87 (10.81) | 57.28 (10.71) | 56.39 (10.57) | <0.001 |
| Calcium, mg/dL | 9.40 (9.20,9.60) | 9.40 (9.20,9.60) | 9.40 (9.20,9.60) | 9.40 (9.20,9.60) | 9.40 (9.20,9.60) | 0.038 |
| Phosphorus, mg/dL | 3.70 (3.40,4.10) | 3.70 (3.40,4.10) | 3.70 (3.40,4.10) | 3.70 (3.30,4.10) | 3.70 (3.40,4.10) | 0.865 |
| Gender |  |  |  |  |  | <0.001 |
| Male | 6578 (48.41%) | 1752 (51.57%) | 1753 (51.63%) | 1610 (47.37%) | 1463 (43.05%) |  |
| Female | 7011 (51.59%) | 1645 (48.43%) | 1642 (48.37%) | 1789 (52.63%) | 1935 (56.95%) |  |
| Race |  |  |  |  |  | <0.001 |
| Mexican American | 2164 (15.92%) | 554 (16.31%) | 533 (15.70%) | 551 (16.21%) | 526 (15.48%) |  |
| Other Hispanic | 1563 (11.50%) | 347 (10.21%) | 377 (11.10%) | 406 (11.94%) | 433 (12.74%) |  |
| Non-Hispanic White | 5842 (42.99%) | 1529 (45.01%) | 1572 (46.30%) | 1487 (43.75%) | 1254 (36.90%) |  |
| Non-Hispanic Black | 2679 (19.71%) | 683 (20.11%) | 560 (16.49%) | 574 (16.89%) | 862 (25.37%) |  |
| Other Race | 1341 (9.87%) | 284 (8.36%) | 353 (10.40%) | 381 (11.21%) | 323 (9.51%) |  |
| Income-poverty Ratio |  |  |  |  |  | 0.386 |
| Low | 3817 (30.61%) | 985 (31.82%) | 949 (30.40%) | 937 (29.78%) | 946 (30.48%) |  |
| Middle | 4472 (35.87%) | 1123 (36.27%) | 1105 (35.39%) | 1124 (35.73%) | 1120 (36.08%) |  |
| High | 4179 (33.52%) | 988 (31.91%) | 1068 (34.21%) | 1085 (34.49%) | 1038 (33.44%) |  |
| Education level |  |  |  |  |  | 0.006 |
| Less than high school | 3537 (26.73%) | 892 (27.01%) | 877 (26.51%) | 871 (26.19%) | 897 (27.20%) |  |
| High school | 2949 (22.28%) | 799 (24.20%) | 711 (21.49%) | 766 (23.03%) | 673 (20.41%) |  |
| Above high school | 6748 (50.99%) | 1611 (48.79%) | 1720 (52.00%) | 1689 (50.78%) | 1728 (52.40%) |  |
| Marital |  |  |  |  |  | 0.162 |
| Married | 8857 (65.20%) | 2230 (65.67%) | 2244 (66.16%) | 2219 (65.28%) | 2164 (63.68%) |  |
| Living alone | 4728 (34.80%) | 1166 (34.33%) | 1148 (33.84%) | 1180 (34.72%) | 1234 (36.32%) |  |
| BMI |  |  |  |  |  | 0.009 |
| Normal weight | 3215 (23.89%) | 737 (21.94%) | 791 (23.58%) | 811 (24.05%) | 876 (25.98%) |  |
| Over-weight | 4711 (35.01%) | 1210 (36.02%) | 1197 (35.68%) | 1183 (35.08%) | 1121 (33.24%) |  |
| Obese | 5532 (41.11%) | 1412 (42.04%) | 1367 (40.75%) | 1378 (40.87%) | 1375 (40.78%) |  |
| CAD score |  |  |  |  |  | <0.001 |
| None | 5838 (44.40%) | 1301 (39.47%) | 1505 (45.98%) | 1539 (46.82%) | 1493 (45.32%) |  |
| Slight | 3939 (29.95%) | 1066 (32.34%) | 957 (29.24%) | 956 (29.08%) | 960 (29.14%) |  |
| Heavy | 3373 (25.65%) | 929 (28.19%) | 811 (24.78%) | 792 (24.09%) | 841 (25.53%) |  |
| Diabetes |  |  |  |  |  | 0.003 |
| No | 10647(78.35%) | 2635 (77.57%) | 2708 (79.76%) | 2702 (79.49%) | 2602 (76.57%) |  |
| Yes | 2942 (21.65%) | 762 (22.43%) | 687 (20.24%) | 697 (20.51%) | 796 (23.43%) |  |
| Smoking |  |  |  |  |  | <0.001 |
| Never | 6990 (51.47%) | 1550 (45.68%) | 1684 (49.62%) | 1813 (53.35%) | 1943 (57.20%) |  |
| Former | 4345 (31.99%) | 1209 (35.63%) | 1120 (33.00%) | 1048 (30.84%) | 968 (28.50%) |  |
| Current | 2247 (16.54%) | 634 (18.69%) | 590 (17.38%) | 537 (15.80%) | 486 (14.31%) |  |
| Alcohol intake |  |  |  |  |  | <0.001 |
| None | 9815 (76.57%) | 2328 (72.28%) | 2370 (73.99%) | 2505 (78.55%) | 2612 (81.50%) |  |
| Moderate | 1052 (8.21%) | 270 (8.38%) | 282 (8.80%) | 247 (7.75%) | 253 (7.89%) |  |
| Heavy | 1951 (15.22%) | 623 (19.34%) | 551 (17.20%) | 437 (13.70%) | 340 (10.61%) |  |
| Physical activity |  |  |  |  |  | 0.091 |
| Less than moderate | 7723 (56.89%) | 1998 (58.87%) | 1872 (55.24%) | 1907 (56.14%) | 1946 (57.30%) |  |
| Moderate | 1197 (8.82%) | 279 (8.22%) | 306 (9.03%) | 315 (9.27%) | 297 (8.75%) |  |
| Vigorous | 4656 (34.30%) | 1117 (32.91%) | 1211 (35.73%) | 1175 (34.59%) | 1153 (33.95%) |  |
| Klotho, pg/mL | 803.10 (655.60,994.40) | 557.40 (491.00,612.00) | 728.00 (691.80,764.25) | 888.00 (842.60,936.10) | 1172.55 (1071.12,1341.68) | - |
| eGFR, mL/min/1.73 m^2^ | 89.23 (74.4,101.46) | 85.31 (68.80,99.07) | 88.78 (73.69,100.50) | 90.04 (76.09,101.83) | 92.39 (79.13,104.17) | <0.001 |
| ACR |  |  |  |  |  | <0.001 |
| < 30 mg/g | 11688(86.01%) | 2835 (83.46%) | 2913 (85.80%) | 3000 (88.26%) | 2940 (86.52%) |  |
| ≧ 30 mg/g | 1901 (13.99%) | 562 (16.54%) | 482 (14.20%) | 399 (11.74%) | 458 (13.48%) |  |
| CKD |  |  |  |  |  | <0.001 |
| Without CKD | 10856 (79.89%) | 2517 (74.09%) | 2708 (79.76%) | 2829 (83.23%) | 2802 (82.46%) |  |
| Stage 1 | 740 (5.45%) | 157 (4.62%) | 177 (5.21%) | 168 (4.94%) | 238 (7.00%) |  |
| Stage 2 | 715 (5.26%) | 213 (6.27%) | 186 (5.48%) | 161 (4.74%) | 155 (4.56%) |  |
| Stage 3 | 1146 (8.43%) | 437 (12.86%) | 292 (8.60%) | 228 (6.71%) | 189 (5.56%) |  |
| Stage 4 | 93 (0.68%) | 46 (1.35%) | 27 (0.80%) | 11 (0.32%) | 9 (0.26%) |  |
| Stage 5 | 39 (0.29%) | 27 (0.79%) | 5 (0.15%) | 2 (0.06%) | 5 (0.15%) |  |

a Normal continuous variables were presented with mean (SD), while non-normal continuous variables were presented with median (lower quartile, upper quartile). Categorical variables were described using numbers (percentage)

b P values of differences among different TT tertiles were calculated by Kruscal Whallis H test (continuous variables) or chi-square test (categorical variables). If the categorical variables had a theoretical number less than 10%, Fisher’s precision probability test were applied.

Abbr. BMI, body mass index; CAD score, the cardiovascular disease score; eGFR, estimated glomerular filtration rate; ACR, albumin-creatinine ratio; CKD, chronic kidney disease.

Supplementary Table 2, Baseline Characteristics of Participants Regarding to Age^a^

|  | Age groups | | | | p value^b^ |
| --- | --- | --- | --- | --- | --- |
|  | Q1 | Q2 | Q3 | Q4 |  |
| Number of subjects | 3081 | 3415 | 3477 | 3616 |  |
| Age^c^ | 40.00-47.00 | 48.00-56.00 | 57.00-65.00 | 66.00-79.00 | - |
| Calcium, mg/dL | 9.30 (9.10,9.60) | 9.40 (9.20,9.60) | 9.40 (9.20,9.60) | 9.40 (9.20,9.70) | <0.001 |
| Phosphorus, mg/dL | 3.70 (3.30,4.00) | 3.70 (3.40,4.10) | 3.70 (3.40,4.10) | 3.70 (3.35,4.05) | <0.001 |
| Gender |  |  |  |  | <0.001 |
| Male | 1394 (45.25%) | 1672 (48.96%) | 1717 (49.38%) | 1795 (49.64%) |  |
| Female | 1687 (54.75%) | 1743 (51.04%) | 1760 (50.62%) | 1821 (50.36%) |  |
| Race |  |  |  |  | <0.001 |
| Mexican American | 563 (18.27%) | 557 (16.31%) | 584 (16.80%) | 460 (12.72%) |  |
| Other Hispanic | 329 (10.68%) | 407 (11.92%) | 455 (13.09%) | 372 (10.29%) |  |
| Non-Hispanic White | 1247 (40.47%) | 1404 (41.11%) | 1320 (37.96%) | 1871 (51.74%) |  |
| Non-Hispanic Black | 567 (18.40%) | 681 (19.94%) | 796 (22.89%) | 635 (17.56%) |  |
| Other Race | 375 (12.17%) | 366 (10.72%) | 322 (9.26%) | 278 (7.69%) |  |
| Income-poverty Ratio |  |  |  |  | <0.001 |
| Low | 890 (31.03%) | 945 (30.10%) | 941 (29.68%) | 1041 (31.64%) |  |
| Middle | 1006 (35.08%) | 1010 (32.17%) | 1084 (34.20%) | 1372 (41.70%) |  |
| High | 972 (33.89%) | 1185 (37.74%) | 1145 (36.12%) | 877 (26.66%) |  |
| Education level |  |  |  |  | <0.001 |
| Less than high school | 753 (25.18%) | 854 (25.85%) | 857 (25.26%) | 1073 (30.26%) |  |
| High school | 619 (20.70%) | 704 (21.31%) | 818 (24.11%) | 808 (22.79%) |  |
| Above high school | 1619 (54.13%) | 1746 (52.85%) | 1718 (50.63%) | 1665 (46.95%) |  |
| Marital |  |  |  |  | <0.001 |
| Married | 2197 (71.31%) | 2213 (64.84%) | 2239 (64.41%) | 2208 (61.08%) |  |
| Living alone | 884 (28.69%) | 1200 (35.16%) | 1237 (35.59%) | 1407 (38.92%) |  |
| BMI |  |  |  |  | 0.019 |
| Normal weight | 782 (25.56%) | 814 (24.00%) | 792 (23.00%) | 827 (23.21%) |  |
| Over-weight | 1052 (34.39%) | 1145 (33.76%) | 1199 (34.81%) | 1315 (36.91%) |  |
| Obese | 1225 (40.05%) | 1433 (42.25%) | 1453 (42.19%) | 1421 (39.88%) |  |
| CAD score |  |  |  |  | <0.001 |
| None | 1988 (65.94%) | 1751 (52.66%) | 1237 (37.04%) | 862 (24.84%) |  |
| Slight | 706 (23.42%) | 896 (26.95%) | 1100 (32.93%) | 1237 (35.65%) |  |
| Heavy | 321 (10.65%) | 678 (20.39%) | 1003 (30.03%) | 1371 (39.51%) |  |
| Diabetes |  |  |  |  | <0.001 |
| No | 2751 (89.29%) | 2795 (81.84%) | 2564 (73.74%) | 2537 (70.16%) |  |
| Yes | 330 (10.71%) | 620 (18.16%) | 913 (26.26%) | 1079 (29.84%) |  |
| Smoking |  |  |  |  | <0.001 |
| Never | 1825 (59.25%) | 1751 (51.27%) | 1725 (49.64%) | 1689 (46.76%) |  |
| Former | 658 (21.36%) | 941 (27.55%) | 1194 (34.36%) | 1552 (42.97%) |  |
| Current | 597 (19.38%) | 723 (21.17%) | 556 (16.00%) | 371 (10.27%) |  |
| Alcohol intake |  |  |  |  | <0.001 |
| None | 2101 (72.77%) | 2406 (74.54%) | 2520 (76.76%) | 2788 (81.52%) |  |
| Moderate | 223 (7.72%) | 246 (7.62%) | 288 (8.77%) | 295 (8.63%) |  |
| Heavy | 563 (19.50%) | 576 (17.84%) | 475 (14.47%) | 337 (9.85%) |  |
| Physical activity |  |  |  |  | <0.001 |
| Less than moderate | 1572 (51.12%) | 1896 (55.57%) | 2062 (59.36%) | 2193 (60.66%) |  |
| Moderate | 319 (10.37%) | 323 (9.47%) | 273 (7.86%) | 282 (7.80%) |  |
| Vigorous | 1184 (38.50%) | 1193 (34.96%) | 1139 (32.79%) | 1140 (31.54%) |  |
| Klotho, pg/mL | 826.30 (674.20,1030.30) | 819.70 (671.80,1009.45) | 799.20 (655.30,999.20) | 770.05 (628.55,952.65) | <0.001 |
| eGFR, mL/min/1.73 m^2^ | 103.67 (91.19,110.85) | 96.25 (82.76,103.96) | 87.10 (73.95,96.49) | 73.67 (60.63,86.65) | <0.001 |
| ACR |  |  |  |  | <0.001 |
| < 30 mg/g | 2797 (90.78%) | 3047 (89.22%) | 2968 (85.36%) | 2876 (79.54%) |  |
| ≧ 30 mg/g | 284 (9.22%) | 368 (10.78%) | 509 (14.64%) | 740 (20.46%) |  |
| CKD |  |  |  |  | <0.001 |
| Without CKD | 2779 (90.20%) | 2971 (87.00%) | 2798 (80.47%) | 2308 (63.83%) |  |
| Stage 1 | 220 (7.14%) | 221 (6.47%) | 210 (6.04%) | 89 (2.46%) |  |
| Stage 2 | 48 (1.56%) | 108 (3.16%) | 205 (5.90%) | 354 (9.79%) |  |
| Stage 3 | 22 (0.71%) | 103 (3.02%) | 235 (6.76%) | 786 (21.74%) |  |
| Stage 4 | 8 (0.26%) | 5 (0.15%) | 16 (0.46%) | 64 (1.77%) |  |
| Stage 5 | 4 (0.13%) | 7 (0.20%) | 13 (0.37%) | 15 (0.41%) |  |

a Normal continuous variables were presented with mean (SD), while non-normal continuous variables were presented with median (lower quartile, upper quartile). Categorical variables were described using numbers (percentage)

b P values of differences among different TT tertiles were calculated by Kruscal Whallis H test (continuous variables) or chi-square test (categorical variables). If the categorical variables had a theoretical number less than 10%, Fisher’s precision probability test were applied.

c Age was presented as Min – Max to show the range of each group.

Abbr. BMI, body mass index; CAD score, the cardiovascular disease score; eGFR, estimated glomerular filtration rate; ACR, albumin-creatinine ratio; CKD, chronic kidney disease.

Supplementary Table 3, Baseline Characteristics of Participants Regarding to Gender^a^

|  | Gender | | p value^b^ |
| --- | --- | --- | --- |
|  | Male | Female |  |
| Number of subjects | 6578 | 7011 |  |
| Age | 57.98 (10.81) | 57.33 (10.85) | <0.001 |
| Calcium, mg/dL | 9.40 (9.20,9.60) | 9.40 (9.20,9.60) | 0.044 |
| Phosphorus, mg/dL | 3.60 (3.20,3.90) | 3.80 (3.50,4.20) | <0.001 |
| Race |  |  | 0.029 |
| Mexican American | 1033 (15.70%) | 1131 (16.13%) |  |
| Other Hispanic | 701 (10.66%) | 862 (12.29%) |  |
| Non-Hispanic White | 2882 (43.81%) | 2960 (42.22%) |  |
| Non-Hispanic Black | 1301 (19.78%) | 1378 (19.65%) |  |
| Other Race | 661 (10.05%) | 680 (9.70%) |  |
| Income-poverty Ratio |  |  | <0.001 |
| Low | 1761 (29.05%) | 2056 (32.10%) |  |
| Middle | 2147 (35.41%) | 2325 (36.30%) |  |
| High | 2155 (35.54%) | 2024 (31.60%) |  |
| Education level |  |  | 0.462 |
| Less than high school | 1734 (26.87%) | 1803 (26.59%) |  |
| High school | 1462 (22.66%) | 1487 (21.93%) |  |
| Above high school | 3257 (50.47%) | 3491 (51.48%) |  |
| Marital |  |  | <0.001 |
| Married | 4730 (71.92%) | 4127 (58.89%) |  |
| Living alone | 1847 (28.08%) | 2881 (41.11%) |  |
| BMI |  |  | <0.001 |
| Normal weight | 1446 (22.22%) | 1769 (25.46%) |  |
| Over-weight | 2653 (40.76%) | 2058 (29.62%) |  |
| Obese | 2410 (37.03%) | 3122 (44.93%) |  |
| CAD score |  |  | <0.001 |
| None | 2778 (43.78%) | 3060 (44.97%) |  |
| Slight | 1808 (28.49%) | 2131 (31.32%) |  |
| Heavy | 1759 (27.72%) | 1614 (23.72%) |  |
| Diabetes |  |  | <0.001 |
| No | 5068 (77.04%) | 5579 (79.57%) |  |
| Yes | 1510 (22.96%) | 1432 (20.43%) |  |
| Smoking |  |  | <0.001 |
| Never | 2706 (41.16%) | 4284 (61.13%) |  |
| Former | 2631 (40.02%) | 1714 (24.46%) |  |
| Current | 1237 (18.82%) | 1010 (14.41%) |  |
| Alcohol intake |  |  | <0.001 |
| None | 4364 (70.22%) | 5451 (82.55%) |  |
| Moderate | 760 (12.23%) | 292 (4.42%) |  |
| Heavy | 1091 (17.55%) | 860 (13.02%) |  |
| Physical activity |  |  | 0.001 |
| Less than moderate | 3632 (55.29%) | 4091 (58.38%) |  |
| Moderate | 597 (9.09%) | 600 (8.56%) |  |
| Vigorous | 2340 (35.62%) | 2316 (33.05%) |  |
| Klotho, pg/mL | 784.30 (645.20,965.50) | 822.60 (665.35,1024.00) | <0.001 |
| eGFR, mL/min/1.73 m2 | 88.20  (74.06,99.83) | 90.67 (74.81,103.04) | <0.001 |
| ACR |  |  | 0.01 |
| < 30 mg/g | 5606 (85.22%) | 6082 (86.75%) |  |
| ≧ 30 mg/g | 972 (14.78%) | 929 (13.25%) |  |
| CKD |  |  | <0.001 |
| Without CKD | 5245 (79.74%) | 5611 (80.03%) |  |
| Stage 1 | 331 (5.03%) | 409 (5.83%) |  |
| Stage 2 | 396 (6.02%) | 319 (4.55%) |  |
| Stage 3 | 548 (8.33%) | 598 (8.53%) |  |
| Stage 4 | 35 (0.53%) | 58 (0.83%) |  |
| Stage 5 | 23 (0.35%) | 16 (0.23%) |  |

a Normal continuous variables were presented with mean (SD), while non-normal continuous variables were presented with median (lower quartile, upper quartile). Categorical variables were described using numbers (percentage)

b P values of differences among different TT tertiles were calculated by Kruscal Whallis H test (continuous variables) or chi-square test (categorical variables). If the categorical variables had a theoretical number less than 10%, Fisher’s precision probability test were applied.

Abbr. BMI, body mass index; CAD score, the cardiovascular disease score; eGFR, estimated glomerular filtration rate; ACR, albumin-creatinine ratio; CKD, chronic kidney disease.

Supplementary Table 4, Baseline Characteristics of Participants Regarding to Races^a^

|  | Klotho groups | | | | | p value^b^ |
| --- | --- | --- | --- | --- | --- | --- |
|  | Mexican American | Other Hispanic | Non-Hispanic White | Non-Hispanic Black | Other Race |  |
| Number of subjects | 2164 | 1563 | 5842 | 2679 | 1341 |  |
| Age | 56.23 (10.43) | 57.29 (10.17) | 58.74 (11.35) | 57.56 (10.30) | 55.72 (10.42) | <0.001 |
| Calcium, mg/dL | 9.30 (9.10,9.50) | 9.40 (9.20,9.60) | 9.40 (9.20,9.60) | 9.40 (9.20,9.70) | 9.30 (9.10,9.60) | <0.001 |
| Phosphorus, mg/dL | 3.70 (3.30,4.00) | 3.70 (3.40,4.10) | 3.70 (3.40,4.10) | 3.60 (3.30,4.00) | 3.70 (3.40,4.10) | <0.001 |
| Gender |  |  |  |  |  | 0.029 |
| Male | 1033 (47.74%) | 701 (44.85%) | 2882 (49.33%) | 1301 (48.56%) | 661 (49.29%) |  |
| Female | 1131 (52.26%) | 862 (55.15%) | 2960 (50.67%) | 1378 (51.44%) | 680 (50.71%) |  |
| Income-poverty Ratio |  |  |  |  |  | <0.001 |
| Low | 839 (44.20%) | 512 (37.84%) | 1357 (24.53%) | 772 (31.33%) | 337 (27.60%) |  |
| Middle | 741 (39.04%) | 523 (38.65%) | 1810 (32.72%) | 1002 (40.67%) | 396 (32.43%) |  |
| High | 318 (16.75%) | 318 (23.50%) | 2365 (42.75%) | 690 (28.00%) | 488 (39.97%) |  |
| Education level |  |  |  |  |  | <0.001 |
| Less than high school | 1131 (53.81%) | 559 (37.09%) | 935 (16.32%) | 682 (26.08%) | 230 (17.94%) |  |
| High school | 389 (18.51%) | 300 (19.91%) | 1385 (24.18%) | 670 (25.62%) | 205 (15.99%) |  |
| Above high school | 582 (27.69%) | 648 (43.00%) | 3408 (59.50%) | 1263 (48.30%) | 847 (66.07%) |  |
| Marital |  |  |  |  |  | <0.001 |
| Married | 1519 (70.19%) | 1017 (65.11%) | 3943 (67.51%) | 1355 (50.60%) | 1023 (76.34%) |  |
| Living alone | 645 (29.81%) | 545 (34.89%) | 1898 (32.49%) | 1323 (49.40%) | 317 (23.66%) |  |
| BMI |  |  |  |  |  | <0.001 |
| Normal weight | 290 (13.52%) | 312 (20.09%) | 1423 (24.62%) | 538 (20.27%) | 652 (49.21%) |  |
| Over-weight | 848 (39.53%) | 582 (37.48%) | 2023 (34.99%) | 822 (30.97%) | 436 (32.91%) |  |
| Obese | 1007 (46.95%) | 659 (42.43%) | 2335 (40.39%) | 1294 (48.76%) | 237 (17.89%) |  |
| CAD score |  |  |  |  |  | <0.001 |
| None | 967 (46.38%) | 669 (44.01%) | 2648 (46.75%) | 890 (34.40%) | 664 (51.31%) |  |
| Slight | 537 (25.76%) | 445 (29.28%) | 1727 (30.49%) | 897 (34.67%) | 333 (25.73%) |  |
| Heavy | 581 (27.87%) | 406 (26.71%) | 1289 (22.76%) | 800 (30.92%) | 297 (22.95%) |  |
| Diabetes |  |  |  |  |  | <0.001 |
| No | 1550 (71.63%) | 1184 (75.75%) | 4904 (83.94%) | 1959 (73.12%) | 1050 (78.30%) |  |
| Yes | 614 (28.37%) | 379 (24.25%) | 938 (16.06%) | 720 (26.88%) | 291 (21.70%) |  |
| Smoking |  |  |  |  |  | <0.001 |
| Never | 1238 (57.21%) | 892 (57.11%) | 2632 (45.07%) | 1347 (50.34%) | 881 (65.75%) |  |
| Former | 694 (32.07%) | 496 (31.75%) | 2063 (35.33%) | 776 (29.00%) | 316 (23.58%) |  |
| Current | 232 (10.72%) | 174 (11.14%) | 1145 (19.61%) | 553 (20.67%) | 143 (10.67%) |  |
| Alcohol intake |  |  |  |  |  | <0.001 |
| None | 1659 (81.16%) | 1177 (80.23%) | 4111 (72.68%) | 1950 (77.08%) | 918 (81.89%) |  |
| Moderate | 142 (6.95%) | 122 (8.32%) | 494 (8.73%) | 186 (7.35%) | 108 (9.63%) |  |
| Heavy | 243 (11.89%) | 168 (11.45%) | 1051 (18.58%) | 394 (15.57%) | 95 (8.47%) |  |
| Physical activity |  |  |  |  |  | <0.001 |
| Less than moderate | 1397 (64.68%) | 1002 (64.11%) | 3080 (52.78%) | 1573 (58.76%) | 671 (50.04%) |  |
| Moderate | 165 (7.64%) | 108 (6.91%) | 515 (8.83%) | 264 (9.86%) | 145 (10.81%) |  |
| Vigorous | 598 (27.69%) | 453 (28.98%) | 2240 (38.39%) | 840 (31.38%) | 525 (39.15%) |  |
| Klotho, pg/mL^c^ | 801.20 (652.45,985.73) | 830.90 (672.15,1027.65) | 786.10 (647.40,960.68) | 831.30 (653.00,1081.40) | 819.30 (679.70,985.60) | <0.001 |
| eGFR, mL/min/1.73 m^2^ | 95.90 (82.94,106.18) | 92.14 (78.84,101.66) | 84.94 (70.72,96.35) | 89.93 (73.91,105.92) | 94.05 (80.08,104.35) | <0.001 |
| ACR |  |  |  |  |  | <0.001 |
| < 30 mg/g | 1820 (84.10%) | 1348 (86.24%) | 5159 (88.31%) | 2203 (82.23%) | 1158 (86.35%) |  |
| ≧ 30 mg/g | 344 (15.90%) | 215 (13.76%) | 683 (11.69%) | 476 (17.77%) | 183 (13.65%) |  |
| CKD |  |  |  |  |  | <0.001 |
| Without CKD | 1751 (80.91%) | 1287 (82.34%) | 4673 (79.99%) | 2028 (75.70%) | 1117 (83.30%) |  |
| Stage 1 | 176 (8.13%) | 92 (5.89%) | 199 (3.41%) | 192 (7.17%) | 81 (6.04%) |  |
| Stage 2 | 104 (4.81%) | 83 (5.31%) | 297 (5.08%) | 164 (6.12%) | 67 (5.00%) |  |
| Stage 3 | 111 (5.13%) | 90 (5.76%) | 629 (10.77%) | 249 (9.29%) | 67 (5.00%) |  |
| Stage 4 | 15 (0.69%) | 8 (0.51%) | 35 (0.60%) | 29 (1.08%) | 6 (0.45%) |  |
| Stage 5 | 7 (0.32%) | 3 (0.19%) | 9 (0.15%) | 17 (0.63%) | 3 (0.22%) |  |

a Continuous variables are presented as mean (SD) for continuous variables and numbers (proportion) for categorical variables.

b P values of differences among different TT tertiles were calculated by Kruscal Whallis H test (continuous variables) or chi-square test (categorical variables). If the categorical variables had a theoretical number less than 10%, Fisher’s precision probability test were applied.

c S-Klotho concentration is presented in median, upper and lower quartiles due to its highly skew distribution.

Abbr. BMI, body mass index; CAD score, the cardiovascular disease score; eGFR, estimated glomerular filtration rate; ACR, albumin-creatinine ratio; CKD, chronic kidney disease.

|  | OR, 95% CI | p values |
| --- | --- | --- |
| Independent variable | Klotho after logarithmic transformed | |
| Dependent variable | eGFR, mL/min/1.73 m2 | |
| CKD stage |  |  |
| Without CKD | 2.02 (1.26, 2.78) | <0.001 |
| CKD stage 1 | 1.28 (-0.28, 2.83) | 0.108 |
| CKD stage 2 | 2.87 (0.94, 4.79) | 0.004 |
| CKD stage 3 | 2.03 (0.62, 3.44) | 0.005 |
| CKD stage 4 | 2.71 (-0.15, 5.58) | 0.068 |
| CKD stage 5 | -1.05 (-4.27, 2.17) | 0.533 |

Supplementary Table 5, Association between Serum Soluble Klotho and eGFR in Groups Classified by CKD Condition^a^

1. All models were adjusted for age, gender, race, ratio of income-poverty, educational levels, marital status, BMI, CAD score, smoking, alcohol, physical activity, serum calcium, and serum phosphorus.

Abbr.: OR, odds ration; 95% CI, 95% confidential interval; eGFR, estimated glomerular filtration rate; CKD, chronic kidney disease

Supplementary Table 6, Association between Serum Soluble Klotho and eGFR calculated with MDRD equation

| Outcomes | Crude Model | | Model I^a^ | | Model II^b^ | |
| --- | --- | --- | --- | --- | --- | --- |
|  | β/OR ,95%CI | p values | β/OR ,95%CI | p values | β/OR ,95%CI | p values |
| eGFR, mL/min/1.73m2 | 7.78 (6.46,9.11) | <0.001 | 5.64 (4.41,6.87) | <0.001 | 5.91 (4.63,7.18) | <0.001 |
| CKD stage ≧ 1 | 0.52 (0.43,0.62) | <0.001 | 0.58 (0.48,0.70) | <0.001 | 0.59 (0.48,0.72) | <0.001 |
| CKD stage ≧ 3 | 0.31 (0.24,0.39) | <0.001 | 0.35 (0.27,0.45) | <0.001 | 0.34 (0.26,0.44) | <0.001 |

^a^ Model I: adjusted for age, gender, race, ratio of income-poverty, educational levels, and marital status.

^b^ Model II: adjusted for age, gender, race, ratio of income-poverty, educational levels, marital status, BMI, CAD score, smoking, alcohol, physical activity, serum calcium, and serum phosphorus.

Abbr.: MDRD equation: the Modification of Diet in Renal Disease equation; OR, odds ration; 95% CI, 95% confidential interval; eGFR, estimated glomerular filtration rate; CKD, chronic kidney disease
